# Phase 1b trial of anti-VEGF/PDGFR vorolanib combined with immune checkpoint inhibitors in patients with advanced solid tumors

**DOI:** 10.1007/s00280-022-04406-6

**Published:** 2022-03-05

**Authors:** Nusayba A. Bagegni, Haeseong Park, Katlyn Kraft, Maura O-Toole, Feng Gao, Saiama N. Waqar, Lee Ratner, Daniel Morgensztern, Siddhartha Devarakonda, Manik Amin, Maria Q. Baggstrom, Chris Liang, Giovanni Selvaggi, Andrea Wang-Gillam

**Affiliations:** 1 Division of Oncology, Washington University in St. Louis School of Medicine, St Louis, MO USA; 2 Department of Surgery, Washington University in St. Louis School of Medicine, St Louis, MO USA; 3 Division of Hematology/Oncology, Dartmouth Giesel School of Medicine, Hanover, NH USA; 4 Xcovery Holdings, Inc., North Palm Beach, USA

**Keywords:** Vorolanib, Checkpoint inhibitors, Immunotherapy, Pembrolizumab, Nivolumab, Advanced solid tumors

## Abstract

**Purpose:**

Vorolanib is a multi-target tyrosine kinase inhibitor with anti-angiogenic properties. This study aimed to evaluate the tolerability, safety and efficacy of vorolanib when added to checkpoint inhibitors (CPIs) in patients with advanced solid tumors.

**Methods:**

We conducted a phase 1b study of vorolanib (300 or 400 mg orally once daily) plus pembrolizumab or nivolumab using a standard 3 + 3 design to determine the dose-limiting toxicity (DLT), maximum tolerated dose (MTD) and recommended phase 2 dose (RP2D). The endpoints included safety, toxicity and objective response rate, according to Response Evaluation Criteria in Solid Tumors, version 1.1 (RECIST 1.1).

**Results:**

Sixteen patients (9 in pembrolizumab arm, 7 in nivolumab arm) with gastrointestinal or lung cancers were enrolled. All patients had at least 1 treatment-related adverse event (TRAE). The most common TRAEs across all cohorts were lymphopenia (*n* = 7), leukopenia (*n* = 5), fatigue (*n* = 5), and alanine aminotransferase elevation (*n* = 5); most toxicities were grade (G) 1–2. DLTs were reported in 3 patients at vorolanib 400 mg dose level, with G3 aspartate aminotransferase elevation, G3 rectal hemorrhage, and G3 rash. Of 13 total response-evaluable patients, 2 patients had confirmed partial responses (1 rectal squamous cell cancer and 1 small cell lung cancer). Two patients achieved prolonged stable disease. Vorolanib 300 mg daily was determined to be the RP2D for either pembrolizumab or nivolumab.

**Conclusion:**

Combination vorolanib 300 mg orally once daily plus CPI appears to be a feasible regimen with manageable toxicity and promising efficacy in select tumor types. NCT03511222. Date of Registration: April 18, 2018.

## Introduction

Immune checkpoint inhibitors (CPIs) have emerged as a standard treatment option for a multitude of advanced solid malignancies, including gastrointestinal (GI) and pulmonary cancers, either in the front-line or refractory settings. Multi-modality immunotherapy with programmed cell death-1/programmed cell death ligand-1 (PD-1/PD-L1) inhibition plus tyrosine kinase inhibitors (TKIs) or cytotoxic T-lymphocyte-associated protein 4 (CTLA-4) inhibitors have become part of the established therapeutic landscape for multiple tumor types. Pembrolizumab has received FDA approval for the treatment of several solid tumors. Recently approved indications for pembrolizumab include treatment in combination with platinum- and fluoropyrimidine-based chemotherapy for patients with metastatic or locally advanced esophageal or gastroesophageal junction (GEJ) carcinoma, regardless of PD-L1 status [1, 2]. Nivolumab received accelerated approval for the treatment of patients with advanced gastric, esophageal, or GEJ adenocarcinoma in combination with platinum- and fluoropyrimidine-based chemotherapy [3]. In addition, anti-PD-1/PD-L1 inhibitors are now standard therapies in the first-line setting for metastatic non-small cell lung cancers (NSCLC) [4–9], in combination with cytotoxic therapies or CTLA-4 inhibitors, as well as in small cell lung cancer (SCLC). Until recently, both pembrolizumab and nivolumab were approved for the treatment of SCLC [10–12]; FDA approval for nivolumab was subsequently withdrawn [13]. Despite these therapeutic advancements, resistance to combination immune CPIs is inevitable, and patients ultimately succumb to these conditions. Therefore, significant research efforts are focusing on strategies to overcome immunotherapy resistance and further enhance anti-tumor immune response.

Regulation of angiogenesis is a potential mechanism to overcome resistance to CPIs by tumor-mediated immune regulation as well as enhance tumor exposure to other cytotoxic agents. The presence of elevated levels of pro-angiogenic molecules, such as angiopoietin-2, has been associated with immunotherapy resistance and poor prognosis [14]. Vascular endothelial growth factor (VEGF) can inhibit dendritic cell maturation [15] and intra-tumoral T cell trafficking [16], while anti-VEGF therapy can improve T cell infiltration, potentially enhancing response to CPIs [17]. These properties of anti-VEGF therapy may thus improve clinical efficacy and resistance to immune CPIs. In the phase 3 global IMbrave150 trial, bevacizumab, an anti-VEGF monoclonal antibody, in combination with atezolizumab, resulted in improved overall survival (OS) as compared to sorafenib for the first-line treatment of unresectable or metastatic hepatocellular carcinoma (HCC) [18], leading to recent FDA approval for this combination. The IMpower150 trial showed significant improvement in progression-free survival (PFS) and OS with atezolizumab plus bevacizumab combined with carboplatin and paclitaxel compared with standard of care bevacizumab plus carboplatin and paclitaxel in patients with chemotherapy-naïve NSCLC [19]. The randomized phase 2 Lung-MAP S1800A study investigating the role of pembrolizumab plus ramucirumab, an anti-VEGF receptor 2 (VEGFR2) monoclonal antibody, versus chemotherapy in patients with advanced NSCLC post immunotherapy-progression has recently completed enrollment (NCT03971474) [20]. In addition, a phase 1b trial of lenvatinib plus pembrolizumab in unresectable HCC reported promising early efficacy results with an objective response rate (ORR) of 46% and no dose-limiting toxicities (DLT) [21]. A confirmatory phase 3 trial of lenvatinib and pembrolizumab in HCC is ongoing (NCT03713593) [22]. Moreover, although in the phase 3 CLEAR trial, lenvatinib combined with everolimus or pembrolizumab improved PFS in patients with advanced renal cell carcinoma (RCC) as compared to sunitinib, only lenvatinib plus pembrolizumab led to significantly longer OS than sunitinib [23].

Colony-stimulating factor 1 (CSF1)/CSF1 receptor (CSF1R)-mediated signaling is a critical regulator of monocyte/macrophage differentiation, playing a potential role in resistance to CPIs in the preclinical setting. CSF1 promotes tumor-associated macrophages (TAMs) that may undermine anti-tumor immune responses [24]. In fact, the presence of CSF1R + TAMs have been shown to correlate with disease progression and poor prognosis [25]. CSF1R blockade re-programs TAMs and may improve response to or overcome immunotherapy resistance. Consequently, multiple CSF1R inhibitors are under active development, and are being investigated in combination with immunotherapy [26, 27].

Vorolanib (X-82, CM082) is a potent oral TKI against multiple targets including VEGFR, platelet-derived growth factor (PDGFR) and CSF1R, and inhibits angiogenesis. Vorolanib is structurally similar to sunitinib, but designed to improve upon the toxicity profile of this class of therapeutics. Its short half-life of approximately 4–8 h by clinical pharmacokinetic data and limited tissue accumulation allows for continuous dosing [28, 29]. Vorolanib has been investigated in other phase 1/2 trials as monotherapy or in combination with other agents, including chemotherapy with other pathway inhibitors, at doses of 50–800 mg [30]. In these studies, the maximum tolerated dose (MTD) was not reached at 800 mg, but absorption plateaued at 400 to 800 mg daily dosing. Vorolanib was generally well tolerated, with the most common side effects including fatigue, nausea and rash. Vorolanib 300 mg plus everolimus also demonstrated an encouraging efficacy signal in patients with advanced RCC and neuroendocrine tumors [31]. Subsequently, the phase 3 CONCEPT trial of everolimus with or without vorolanib in advanced previously treated RCC showed PFS advantage of vorolanib plus everolimus combination over everolimus alone with median PFS 10.0 versus 6.4 months (HR = 0.70, [95% CI, 0.52–0.94]; *p* = 0.0171), respectively [32].

In light of its favorable toxicity profile as combination therapy, and potential pro-immunogenic and anti-angiogenic properties, we hypothesized that the addition of vorolanib to standard CPIs may augment immunotherapy response and improve clinical efficacy. Therefore, this phase 1b study was designed to determine the safety and MTD of vorolanib in combination with CPIs, pembrolizumab or nivolumab, in patients with advanced solid tumors who were otherwise eligible to receive CPIs per standard of care.

## Methods

### Study design and participants

This study was a single-center, open-label, phase 1b study with a conventional 3 + 3 dose escalation design (NCT03511222) to assess the tolerability, safety and preliminary anti-tumor activity of vorolanib plus CPIs in patients with advanced solid tumors treated at Siteman Cancer Center at Washington University School of Medicine, St Louis, Missouri. Enrollment to both cohorts (vorolanib plus either pembrolizumab or nivolumab) occurred simultaneously; 3 to 6 patients of each cohort were enrolled to receive vorolanib plus a CPI. Vorolanib was administered orally (PO) once daily with food on an outpatient basis at the assigned dose level. The vorolanib dose escalation schedule was set at either 300 mg PO once daily (starting dose, dose level 1) or 400 mg PO daily (dose level 2). Dose level − 1 was to be permitted at vorolanib 200 mg PO once daily if DLT occurred with vorolanib starting dose of 300 mg PO once daily. Vorolanib was given in combination with either standard fixed dose of nivolumab 480 mg intravenously (IV) on an every 28-day cycle or pembrolizumab 200 mg intravenously (IV) on an every 21-day cycle. Dose level advancement did not occur until all patients had completed cycle 1 of each assessed dose level, and the decision to proceed to the next dose level was based on events in the first cycle. Pembrolizumab and nivolumab cohorts were assessed separately. The MTD was defined as the dose level immediately below the dose level at which 2 patients within a cohort dose level (or 2 of 6 patients) experience DLT during the first cycle. Dose escalations continued for both nivolumab and pembrolizumab until the MTD or the highest vorolanib dose level (dose level 2) was achieved.

Eligible patients were at least 18 years or older, with Eastern Cooperative Oncology Group performance status of 0 or 1, and diagnosis of an advanced solid tumor that can be treated with FDA-approved indication for pembrolizumab or nivolumab at the time of study enrollment. Additionally, patients were required to have adequate hematologic, renal and hepatic function, lack of proteinuria, and measurable disease as assessed by Response Evaluation Criteria in Solid Tumor (RECIST) version 1.1 [33]. Patients with uncontrolled intercurrent illness, poorly controlled hypertension, symptomatic arterial peripheral vascular disease or significant cardiovascular disease or condition, prior receipt of CPI, history of clinically significant bleeding, active autoimmune disease or any condition requiring systemic corticosteroid use were excluded. Additional exclusion criteria were the presence of deep vein thrombosis or pulmonary embolism within 4 weeks, clinically evident CNS hemorrhage, and concurrent use of any medications or substances known to be a strong inhibitor or strong inducer of CYP3A4. Patients with treated/stable brain metastases were also eligible.

The trial was approved by the Washington University School of Medicine Institutional Review Board before study initiation, and was performed in accordance with the Declaration of Helsinki, the International Council for Harmonisation guidelines for Good Clinical Practice, the FDA Code of Federal Regulations, the requirements of national drug and data protection laws and other applicable regulatory requirements [34]. All patients were provided written informed consent prior to enrollment. The study was funded by the sponsor, Xcovery Holdings, Inc., which was involved in all aspects of study design, and provided study drug and approved the final version of the manuscript for publication in conjunction with the authors. All authors had full access to all data in the study and provided final approval to submit the manuscript for publication.

### Safety evaluations

Patients must have received at least one dose of vorolanib and one dose of either pembrolizumab or nivolumab to be considered evaluable for safety. Safety evaluations included assessments of adverse events (AEs) and serious adverse events (SAEs). Descriptions and grading scales were defined and reported according to the revised National Cancer Institute Common Terminology Criteria for Adverse Events (NCI CTCAE) version 5.0 [35]. For events with varying severity, the maximum reported grade was utilized in summaries.

A DLT was categorized based on the presence of hematologic and non-hematologic toxicity occurring during the first cycle of study therapy. Hematologic DLT was defined as any of the following that occur during the first cycle that are attributed as possibly, probably, or definitely related to vorolanib: (a) Grade 4 neutropenia of > 7 day duration; (b) Febrile neutropenia of any duration with temperature > 38.5 °C; (c) Grade 4 anemia which requires transfusion therapy on more than two occasions in 7 days; (d) Grade 4 thrombocytopenia which requires transfusion therapy on more than two occasions in 7 days. Non-hematologic DLT was defined as any grade ≥ 3 toxicity that is possibly, probably or definitely related to vorolanib and occurred during the first cycle of therapy, with the following specific exceptions: (a) Grade ≥ 3 nausea which returns to Grade 1 prior to the start of cycle 2; (b) Grade 3 hypertension (BP ≥ 160/100 mmHg) was only considered a DLT if lasting more than 3 days despite optimal intervention. Any vorolanib-related AE that resulted in interruption of pembrolizumab or nivolumab was considered a DLT at the investigator’s discretion, and vorolanib interruption for > 50% of planned dose due to toxicity during the DLT period could have been considered as a DLT. All SAEs were also recorded. Patients removed from study for unacceptable AEs were followed until resolution or stabilization of the event. Following study completion, patients were followed every 2–3 months for 1 year or until death, whichever occurred first.

### Tumor response evaluations

Response assessment by RECIST 1.1 and iRECIST was performed by staging CT or MRI and occurred at baseline and at every 9-week intervals (or 3 cycles) in patients treated with pembrolizumab, or every 8-week intervals (or 2 cycles) in those treated with nivolumab. Patients who had any on-treatment imaging studies were evaluable for tumor response. Treatment beyond progression was permitted per discretion of the treating study investigator. Patients could continue study therapy unless excessive toxicity or clinical or radiographic disease progression confirmed by a subsequent scan per RECIST.

### Statistical considerations

The primary study objective was to determine the recommended phase 2 dose (RP2D) of vorolanib in combination with CPIs. Secondary objective was to assess the safety and toxicity of both combination regimens. The data analyses were descriptive in nature and summary statistics were utilized for demographic and important baseline characteristics. ORR was defined per RECIST as the proportion of patients with either complete or partial response to study therapy. Disease control rate (DCR) was defined as the proportion of patients who achieved either complete or partial response or stable disease to study therapy. Response duration was defined from the time of the first evidence of response until progression. PFS and OS were defined from time of the first dose of vorolanib and pembrolizumab or nivolumab to progression or death (PFS) or death alone (OS). Duration of response and change of tumor were described utilizing a spider plot. The distribution of PFS and OS were estimated by the Kaplan–Meier product limit method. All data analyses were performed using SAS 9.4 [SAS Institutes. Cary, NC].

## Results

### Patient population

**Fig. 1:**
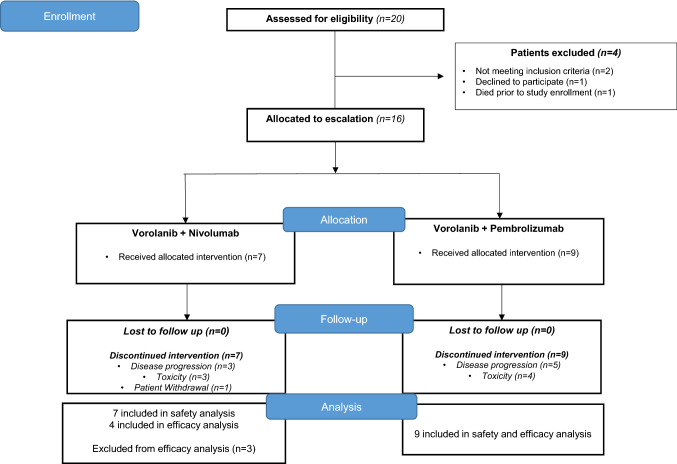
Patient flow diagram

A total of 20 patients were screened for eligibility, 16 patients were enrolled from September 24, 2018 to March 6, 2020 (Fig. 1). Enrolled patients received at least one dose of study therapy. The baseline patient and tumor characteristics are summarized in Table 1. The median age was 65.6 years (range, 45.8–76.4 years), and 43.7% of patients were women (*n* = 7). The majority of patients had advanced GI cancers (*n* = 6, 37.5%); followed by extensive stage SCLC (*n* = 4, 25.0%). Most patients had ECOG performance status of 1 (*n* = 9, 56.2%) and were non-Hispanic Whites (*n* = 14, 87.5%). The median number of prior lines of systemic therapy for all enrolled patients was 1 (range, 0–4). At the time of data cut off (June 1, 2020), the median duration of follow up was 9.6 months (range, 2.0 to 19.4 months). All patients had discontinued study treatment; 5 patients (31.2%) were alive and in follow-up. Eight patients (50.0%) discontinued study treatment due to disease progression; 7 patients (43.7%) discontinued treatment due to toxicity, 1 of whom withdrew enrollment.

**Table 1:** Baseline characteristics of enrolled patients

Characteristics	No. (%)
Age (median, years)	65.6
Sex	
Men	9 (56%)
Women	7 (44%)
Race	
White	14 (88%)
Black	1 (6%)
Asian	1 (6%)
ECOG performance status	
0	7 (44%)
1	9 (56%)
Diagnoses	
Esophageal/GEJ/Gastric	6 (38%)
Small cell lung cancer	4 (25%)
Hepatocellular carcinoma	2 (13%)
Non-small cell lung cancer	1 (6%)
High grade pancreatic NET	1 (6%)
Carcinoid of the lung	1 (6%)
Rectal squamous cell carcinoma	1 (6%)
Prior lines of systemic therapy (median, range)	1, 0–4
0–1 line	11
2–4 lines	5
PD-L1 Status^a^	
PD-L1 positive	5
PD-L1 negative	4
Unknown	7
Mismatch-Repair Status	
Mismatch repair-proficient	10
Unknown	6
Tumor mutational burden status	
Low (< 10 Mut/Mb)	5
Unknown	11

*GEJ* gastroesophageal junction, *NET* neuroendocrine tumor, *Mut/Mb* mutations per megabase

^a^PDL-1 positivity was determined by local testing and defined as either Combined positive score (CPS) ≥ 1, Tumor proportion score (TPS) ≥ 1, or positive by immunohistochemistry (IHC+)

### Safety and tolerability

The 16 enrolled patients received a mean of 4 cycles (range, 1–16) of study therapy across all cohorts and dose levels. Vorolanib plus CPI was well tolerated by most patients, as outlined in Table 2. The most common treatment-related adverse events (TRAE) were lymphopenia (*n* = 7), leukopenia (*n* = 5), fatigue (*n* = 5), neutropenia (*n* = 4), myalgias (*n* = 4) and liver function abnormalities (alanine aminotransferase (ALT) elevation *n* = 5; aspartate aminotransferase (AST) elevation *n* = 4). Most TRAE were grade 1–2 in severity. The most common grade 3 or higher TRAE occurring in more than 1 patient during the course of study therapy included neutropenia (*n* = 4), leukopenia (*n* = 2) and AST elevation (*n* = 2). A total of 3 DLTs were experienced across all dose levels. SAEs were reported in a total of 10 patients (62.5%), 4 patients experienced treatment-related SAEs including grade 3 acute pancreatitis requiring hospitalization following 15 cycles of study therapy (*n* = 1), grade 3 maculopapular rash (*n* = 1, DLT), grade 3 rectal hemorrhage (*n* = 1, DLT) and a patient who developed grade 3 AST and grade 4 ALT elevation following 3 cycles of study therapy. The 6 additional reported SAEs were attributed to other causes not related to study drug.

**Table 2:** Summary of treatment-related adverse events

Variable	Vorolanib + Pembro				Vorolanib + Nivo				All patients (*n* = 16)	
	Vorolanib 300 mg + pembro (*n* = 3)		Vorolanib 400 mg + pembro (*n* = 6)		Vorolanib 300 mg + nivo (*n* = 4)		Vorolanib 400 mg + nivo (*n* = 3)			
	All grades, *n*	Grade 3–4, *n*	All grades, *n*	Grade 3–4, *n*	All grades, *n*	Grade 3–4, *n*	All grades, *n*	Grade 3–4, *n*	All grades, *n* (%)	Grade 3–4, *n* (%)
Lymphopenia			3	1	2		2		7 (43.7%)	1 (6.2%)
Leukopenia			2	1	2	1	1		5 (31.2%)	2 (12.5%)
Anemia					1		1		2 (12.5%)	
Neutropenia			2	2	2	2			4 (25.0%)	4 (25.0%)
Thrombocytopenia			2		1				3 (18.7%)	
Fatigue	2		3						5 (31.2%)	
ALT elevation			4	1	1				5 (31.2%)	1 (6.2%)
AST elevation			3	2*	1				4 (25.0%)	2 (12.5%)
ALP elevation			2	1					2 (12.5%)	1 (6.2%)
Myalgia			3		1				4 (25.0%)	
Dysgeusia			1		1		1		3 (18.7%)	
Hypertension	1		1	1					2 (12.5%)	1 (6.2%)
Proteinuria			3						3 (18.7%)	
Hair color change			2						2 (12.5%)	
Anorexia/weight loss					2				2 (12.5%)	
Diarrhea					1		1	1	2 (12.5%)	1 (6.2%)
Hypothyroidism/TSH elevation			1				1		2 (12.5%)	
Rectal hemorrhage				1*					1 (6.2%)	1 (6.2%)
Pancreatitis or amylase/lipase elevation				1		1			2 (12.5%)	2 (12.5%)
Skin Rash					1		1	1*	2 (12.5%)	1 (6.2%)
Peripheral sensory neuropathy			1		1				2 (12.5%)	
Pruritis			1				1		2 (12.5%)	

Data are number (*n*) and include percent (%) when indicated. Treatment-related adverse events experienced by two or more patients are listed in this table. Dose-limiting toxicities observed in 3 patients are also included and delineated by (*). All adverse events were assessed and reported according to the revised National Cancer Institute Common Terminology Criteria for Adverse Events (NCI CTCAE) version 5.0

*ALP*: alkaline phosphatase elevation, *ALT* alanine aminotransferase, *AST* aspartate aminotransferase, *TSH* thyroid stimulating hormone

A total of three patients were treated at the vorolanib 300 mg PO daily plus pembrolizumab dose level with no observed DLTs. No grade ≥ 3 TRAEs were observed in vorolanib 300 mg plus pembrolizumab arm. One of three patients who had a diagnosis of HCC experienced a DLT at vorolanib 400 mg PO daily plus pembrolizumab dose level of grade 3 AST and alkaline phosphatase elevation that was treated as immune-mediated hepatitis, refractory to corticosteroids. Three additional patients were then enrolled to this dose level, of which 1 additional patient (thus 2 of 6 patients) experienced a DLT of grade 3 rectal hemorrhage. The patient who experienced grade 3 rectal hemorrhage had a diagnosis of rectal squamous cell carcinoma, and this toxicity was also attributed to tumor ulceration in the setting of rivaroxaban use (attributed as possibly related to vorolanib and probably related to disease). This patient ultimately continued on study therapy for a total of 6 cycles due to ongoing clinical benefit. Therefore, vorolanib 300 mg was determined as the RP2D for pembrolizumab combination.

A total of 4 patients were treated at the vorolanib 300 mg PO daily plus nivolumab dose level. One patient withdrew enrollment during cycle 1 due to grade 2 myalgias (which ultimately recovered) and was thereby replaced. No DLTs were experienced at this dose level. Grade 3 or higher TRAEs in the vorolanib 300 mg once daily plus nivolumab arms included: leukopenia (*n* = 1), neutropenia (*n* = 2), elevated serum amylase (*n* = 1), elevated serum lipase (*n* = 1) and oral mucositis (*n* = 1). Three patients were then enrolled into vorolanib 400 mg PO daily plus nivolumab dose level, 1 of 3 patients experienced a DLT of grade 3 rash. MTD for nivolumab combination could not be determined within the scope of this study as no additional patients were enrolled; therefore, vorolanib 300 mg was determined to be the RP2D for nivolumab combination based on tolerability.

In total, 7 out of 16 patients (43.7%) discontinued study therapy due to TRAEs, as outlined in Table 3. One patient treated with vorolanib 300 mg PO daily plus nivolumab required protocol-mandated permanent therapy discontinuation due to prolonged hospitalization for pancreatitis, presumably immune-mediated, although this ultimately resolved without administration of corticosteroids. Two patients treated with 400 mg PO vorolanib plus nivolumab discontinued therapy due to toxicity—one patient developed autoimmune colitis, and the other patient developed grade 3 rash (a DLT). Four patients treated with 400 mg PO vorolanib plus pembrolizumab discontinued therapy due to toxicity—1 with rectal hemorrhage possibly attributed to vorolanib, and 3 patients who developed grade ≥ 3 liver function abnormality.

**Table 3:** Summary of reason for treatment cessation

Reason for treatment cessation	Vorolanib + Pembro		Vorolanib + Nivo		All patients (n = 16)
	Vorolanib 300 mg + pembro (n = 3)	Vorolanib 400 mg + pembro (n = 6)	Vorolanib 300 mg + nivo (n = 4)	Vorolanib 400 mg + nivo (n = 3)	
Disease progression	3	2	2	1	8
Patient discretion	0	0	1	0	1
Adverse event	0	4*	1	2^a^	7

Summary of reasons that subjects discontinued study participation. Dose-liming toxicity (*grade 3 AST elevation (*n* = 1) and ^a^grade 3 rash (*n* = 1))

### Anti-tumor activity

**Fig. 2:**
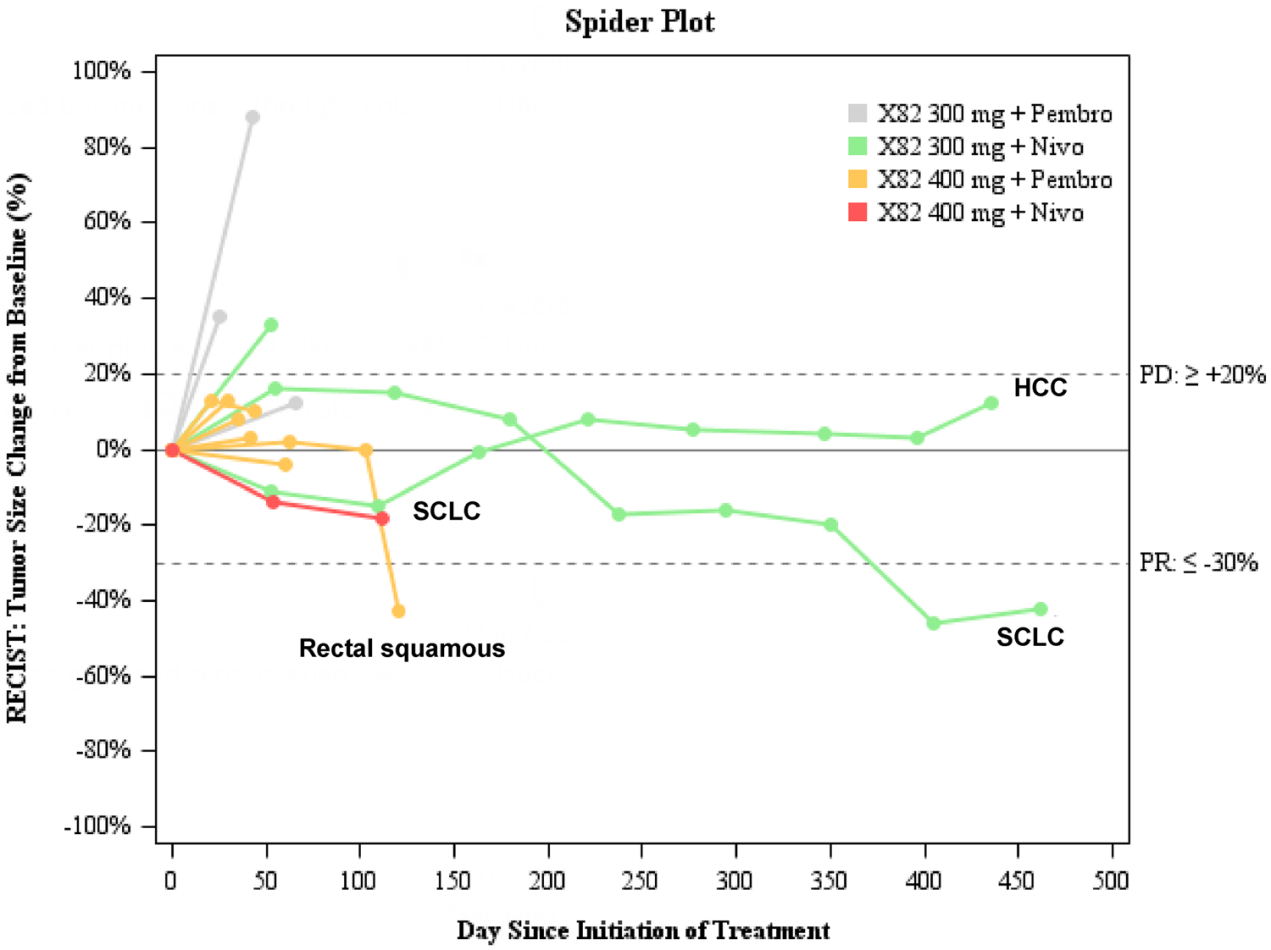
Radiographic tumor response and duration of therapy**.** Spider plot of best overall response. Radiographic response evaluated on the basis of Response Evaluation in Solid Tumors version 1.1 (RECIST v1.1). Each line represents one patient. The dotted lines at + 20% represent cutoffs for progressive disease and at − 30% represent cutoffs for partial response. X82 300 mg: Vorolanib 300 mg. X82 400 mg: Vorolanib 400 mg. Pembro: Pembrolizumab. Nivo: Nivolumab. HCC: Hepatocellular cancer. SCLC: Small cell lung cancer

Three of the 16 enrolled patients were excluded from tumor response assessment due to withdrawal prior to first on-study tumor assessment imaging, therefore, 13 total patients were evaluable for tumor response measurement. Importantly, no differences in tumor response assessment was observed between RECIST and iRECIST criteria. No complete responses were observed. The ORR in the overall study population was 15.4% (*n* = 2 of 13 patients achieved partial response), including one patient with rectal squamous cell carcinoma treated with vorolanib 400 mg plus pembrolizumab, and a second patient with extensive stage SCLC treated with vorolanib 300 mg plus nivolumab. The patient with rectal squamous cell carcinoma was treated beyond initial radiographic progression based on clinical benefit as determined by the treating study investigator, and despite initial grade 3 rectal hemorrhage, as previously described. The overall DCR was 61.5% (*n* = 8 of 13 patients). In Fig. 2, a spider plot illustrates durable responses achieved. This includes 2 patients treated with vorolanib 300 mg plus nivolumab; a patient with extensive stage SCLC achieving durable partial response lasting 15.2 months, and a patient with HCC achieving stable disease lasting 14.3 months. At the time of data cutoff, the median PFS was 6.2 months, and the median OS was 9.6 months. The 95% confidence intervals (CI) were not reported because the upper boundary of the CI could not be estimated due to small sample size.

## Discussion

Our phase 1 dose-finding study of vorolanib in combination with CPIs demonstrates that vorolanib 300 mg once daily plus either pembrolizumab or nivolumab is a well-tolerated regimen with manageable toxicity profile in patients with pretreated, advanced solid tumors. All enrolled patients experienced at least 1 TRAE, however, most toxicities were grade 1–2 in severity. Overall, the most frequent grade 3 or higher TRAEs were manageable, and included leukopenia, neutropenia and AST elevation. No DLTs were observed in the vorolanib 300 mg dose levels. Regarding vorolanib 400 mg arms, 2 DLTs were observed in the vorolanib 400 mg plus pembrolizumab arm, and included grade 3 liver toxicity and grade 3 rectal hemorrhage; one DLT of grade 3 rash was observed in the vorolanib 400 mg plus nivolumab arm. Due to lack of efficacy signal, no additional patients were enrolled.

Although our study is limited by low sample size enrolling a diverse patient population with varying cancer subtypes, no new safety signals were observed with combination vorolanib plus CPIs. Our safety findings aligned with that seen in other studies combining anti-angiogenic agents plus CPIs. A total of 9 out of 16 patients experienced at least 1 or more grade ≥ 3 TRAE, predominately occurring at the 400 mg vorolanib dose levels. Hematologic toxicity was manageable. No therapy-related deaths occurred. Vorolanib resulted in a low number of expected anti-angiogenic therapy related toxicity, including low rates of grade ≥ 3 hypertension (*n* = 1) at vorolanib 400 mg plus pembrolizumab dose level. No thromboembolic events were observed in this study. One patient with rectal squamous cell cancer experienced rectal hemorrhage, which was partially attributed to vorolanib, anticoagulation use, as well as tumor ulceration. Liver enzyme elevation was seen in a proportion of patients, which was attributed to either vorolanib versus CPI.

During the course of this study, new toxicity data became available from a concurrent study of vorolanib plus CPI combination in patients with refractory thoracic tumors conducted at Vanderbilt University (NCT03583086) [36]. Based on this data, it was determined that further enrollment to the vorolanib 400 mg PO daily plus nivolumab combination arm would be discontinued, and the expansion cohort was to enroll 6 additional patients with advanced SCLC at vorolanib 300 mg PO daily plus nivolumab dose level. This supports the RP2D of vorolanib 300 mg as determined by our study. Nevertheless, at the time of the planned study protocol amendment, FDA approvals led to a shift in the first-line treatment paradigm for SCLC to include combination chemo-immunotherapy as standard practice, thus restricting study enrollment capabilities due to limited number of immunotherapy-naïve patients. Therefore, this study was ultimately closed to accrual. More recently, in consulting with the FDA, the manufacturer decided to withdraw the indication for nivolumab for the treatment of SCLC in the U.S. based on results of the confirmatory CheckMate-451 and CheckMate-331 studies, which have since failed to meet primary OS endpoints in this population as either maintenance post first-line chemotherapy or in the second-line setting [11–13].

The overall ORR in the entire study cohort was 15.4% (*n* = 2 of 13 patients). A total of six patients (46.1%) achieved stable disease: 5 patients of whom were treated at vorolanib 400 mg dose level, and 1 treated at vorolanib 300 mg dose level. Notable durable responses were seen in 2 patients; one with HCC achieving stable disease lasting over 12 months, and another with extensive stage SCLC achieving late-onset partial response after 12 months of combination study therapy. Neither of these patients had available molecular tumor tissue testing for mismatch repair status, tumor mutational burden or PD-L1 testing. Thus, it is unclear if these patients could have otherwise achieved such clinical benefit with immunotherapy alone.

Immunotherapy has rapidly transformed the landscape of anti-cancer therapeutics development; however, the majority of patients with advanced cancer do not benefit from single agent immunotherapy [37]. Thus, strategies attempting to expand the success of immunotherapy relies on identifying and targeting key regulators of immune suppression within the tumor microenvironment that could drive primary or acquired resistance to immunotherapy. Proangiogenic factors, including VEGF and angiopoietin-2, are known to promote recruitment of tumor promoting macrophages, and preclinical models illustrate modulation of the tumor vasculature by anti-angiogenic agents may reduce these macrophages and induce immune response by increasing immune effector cell infiltration when combined with immunotherapy [38, 39]. Our study finds that vorolanib is an orally bioavailable multi-kinase inhibitor with favorable toxicity profile when used in combination with CPIs. Nevertheless, identifying these key immunogenic and angiogenic biomarkers to select patients who will in fact derive clinical benefit to guide optimal use of combination anti-angiogenic drugs and immunotherapy remain to be elucidated [40, 41].

In conclusion, our study demonstrates vorolanib at 300 mg plus CPI combination to be a tolerable regimen with potential durable activity in advanced refractory solid tumors, warranting further investigation in a larger patient population. Further biomarker-driven investigation will be required to identify those patients who will achieve optimal response and benefit from combination therapy strategies. ClinicalTrials.gov identifier: NCT03511222.

## Data Availability

The data that support the findings of this study may be available from the corresponding author upon request.
